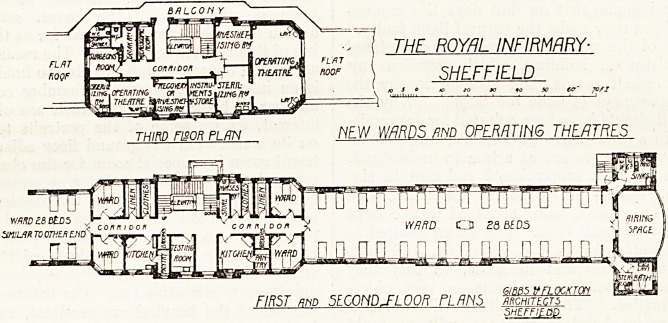# New Units at the Royal Infirmary, Sheffield

**Published:** 1914-02-07

**Authors:** 


### HOSPITAL ARCHITECTURE AND CONSTRUCTION.

### New Units at the Royal Infirmary, Sheffield.

The plans of the new ward block which we have received from the architects, Messrs. Gibbs and Flocton, and which we publish to-day, show the first, second, and third floor plans; but no indication is given of the relative positions of the old building and the new wards. There is a basement under the central part which contains stores and a subway for communication with the old hospital.

The ground, first, and second floors each contains two large wards for twenty-eight beds arranged to some extent on the unit system. Each ward has its own separate ward kitchen, linen, and clothes' store, and two side wards of one bed each, the unit therefore consisting of thirty beds. The large wards are 120 feet long and 27 feet wide. Twenty-four of the beds are "coupled"—*i.e.*, are placed in pairs between two windows. It is somewhat amazing to find this obsolete and thoroughly bad plan adopted in a hospital of such importance as this. It involves the placing of the beds too close together, and is seriously deficient in both the lighting and the ventilation. With the restricted floor space still adhered to by the Local Government Board, the coupled-bed plan is practically a necessity in Poor-Law infirmaries; but there is no excuse for its adoption in the wards of a large general hospital unhampered by the obsolete rules of a Government Department. The sanitary offices are placed in two towers at the angles of the wards, with a wide balcony between.

### The Theatre Unit.

On the third floor the central part is occupied by the operating department. This consists of one large operation theatre evidently planned for two tables, and a smaller theatre for one table. Each theatre has its own anæsthetic and sterilising room. There is also a surgeons' room, with cloak room and w.c., and a microscopic room. The position of the w.c. is an objectionable feature. If the provision of a w.c. in close proximity to the theatre is a necessity, and surgeons are certainly not unanimous on the point, it ought to be so placed that by no possible chance can the air be drawn from it into the corridor leading to the theatres.

### Talbot Ventilation System.

The theatres are ventilated on the Talbot system. This system, the invention of Mr. F. J. Talbot, chairman of Messrs. Ibbotson Brothers and Company, of the Globe Steel Works, Sheffield, consists of an apparatus for washing the air by water sprays.

The essential principle of the system is that the air, which is drawn in by a power-driven fan, in its passage from the outside to the room is washed by water which is violently broken up into a very fine spray, which causes the thorough mixing of the air and water. Every solid particle in the air is thus separated and carried down by the water, and the air leaves the purifier cleansed from all impurities. This is what is claimed for the system, and on the face of it there seems a strong case in its favour. The novel feature is the way in which the water is violently mixed with the air; there being, of course, nothing new in the plan of washing the air by means of a water spray. To warm the incoming air it is passed over a battery of heated coils.

We said before that the unit system had to "some extent" been adopted in the arrangement of the wards. In the arrangement of the operating department the unit system has no place. Instead of each unit having its own theatre and necessary adjuncts, what must be regarded as three theatres with accessories partly used in common are grouped together; and, moreover, the corridor leading to the theatres is the way of access for patients to the flat roof.

It seems a pity that so important an addition to so important a hospital should in many points fall far short of modern standards.

## Figures and Tables

**Figure f1:**